# Intracardiac anatomical relationships and potential for streaming in double inlet left ventricles

**DOI:** 10.1371/journal.pone.0188048

**Published:** 2017-11-30

**Authors:** Sophie L. Meyer, Monique R. Jongbloed, Siew Y. Ho, Margot M. Bartelings, Karen P. McCarthy, Hideki Uemura, Tjark Ebels

**Affiliations:** 1 Department of Cardiothoracic Surgery, University Medical Center Groningen, University of Groningen, Groningen, The Netherlands; 2 Department of Cardiology, Leiden University Medical Center, Leiden University, Leiden, The Netherlands; 3 Department of Anatomy and Embryology, Leiden University Medical Center, Leiden University, Leiden, The Netherlands; 4 Cardiac Morphology Unit, Royal Brompton Hospital, National Heart & Lung Institute, Imperial College London, London, United Kingdom; Indiana University, UNITED STATES

## Abstract

The aim of this study was to gain better understanding of the variable anatomical features of double inlet left ventricle hearts without cavopulmonary connection that would potentially facilitate favorable streaming. Thirty-nine post-mortem specimens of double inlet left ventricle without cavopulmonary connection were investigated. The focus was on anatomical characteristics that could influence the flow and separation of deoxygenated and oxygenated blood in the ventricles. Elements of interest were the ventriculoarterial connection, the spatial relationship of the ventricles, the position and size of the great arteries, the ventricular septal defect, the presence of relative outflow tract stenosis and the relationship of the inflow and outflow tracts. The most common anatomy was a discordant ventriculoarterial connection with an anatomically left-sided morphologically right ventricle (n = 12, 31%). When looking at the pulmonary trunk/aorta ratio, 21 (72%) hearts showed no pulmonary stenosis relative to the aorta. The ventricular septal defect created a relative subpulmonary or subaortic stenosis in 13 (41%) cases. Sixteen (41%) hearts had a parallel relationship of the inflow and outflow tracts, facilitating separation of deoxygenated and oxygenated blood streams. On the other end of the spectrum were 10 (25%) hearts with a perpendicular relationship, which might lead to maximum mixing of the blood streams. The relationship of the inflow and outflow tracts as well as the presence of (sub-) pulmonary stenosis might play a crucial role in the distribution of blood in double inlet left ventricle hearts. Additional in vivo studies will be necessary to confirm this postulation.

## Introduction

Double inlet left ventricle (DILV) is a form of functionally univentricular heart where both the left and the right atrium are connected to the morphologically left ventricle. Usually, the morphologically right ventricle is hypoplastic. The anatomic heterogeneity in the group of DILV hearts is characterized by the position of the ventricles to each other and the relationship of the great arteries to each other as well as to the ventricles [1].

The Fontan procedure is the palliative surgical procedure used in children with functionally univentricular hearts. Modification of the procedure over the years as well as improved perioperative care has led to an improvement of early survival of Fontan patients [2]. However, so far it was not possible to achieve significant improvement of long-term results of the Fontan procedure and median survival stagnates around 30 years after cavopulmonary connection [3,4]. In addition, the mode of decline and death of Fontan patients is dismal: ventricular failure, hepatic cirrhosis,carcinoma and protein losing enteropathy being the leading causes of death [5]. Survival free of such events at 25 years is only 29% [6]. Thus, both life expectancy and quality of life remain compromised in Fontan patients.

In contrast to Fontan results, a small, selected subset of DILV patients have been reported to live beyond their 4^th^ decade without Fontan circulation [7–9]. The inter-individual arterial oxygen saturation and therefore the clinical features amongst DILV patients vary widely due to the numerous anatomical differences and the consequent presence or absence of pulmonary and/or systemic obstruction as well as the differences in systemic and pulmonary vascular resistance. It has been suggested that unpalliated patients depend on a favorable streaming mechanism with unobstructed systemic outflow and moderate to severe pulmonary stenosis in order to live with good functional capacity and relatively few symptoms [10]. Little is known about other factors that make hearts of natural survivors superior to those of Fontan patients regarding function and survival.

Considering the aforementioned data on long-term survival of patients palliated with the Fontan procedure, we are convinced that it is highly relevant to determine further the factors that allow a small selection of patients without cavopulmonary connection to live to a comparatively high age without apparent major complaints.

In this study a series of post mortem specimens without cavopulmonary connection was investigated. The aim was to gain better understanding of the variable anatomical features of DILV hearts that would potentially facilitate favorable streaming, as it has never been done before. The fact that streamline flows as those entering into the ventricle do not have an intrinsic tendency to mix unless forced to by turbulence, is one of the basic principles of fluid dynamics, and serves as a basic paradigm for this study [11]. The focus was on anatomical characteristics that include the arrangements of the inflow and outflow tracts, the position and sizes of the great arteries, and the size and shape of the ventricular septal defect that could influence the flow and separation of deoxygenated and oxygenated blood in DILV hearts.

## Material and methods

**Fig 1:**
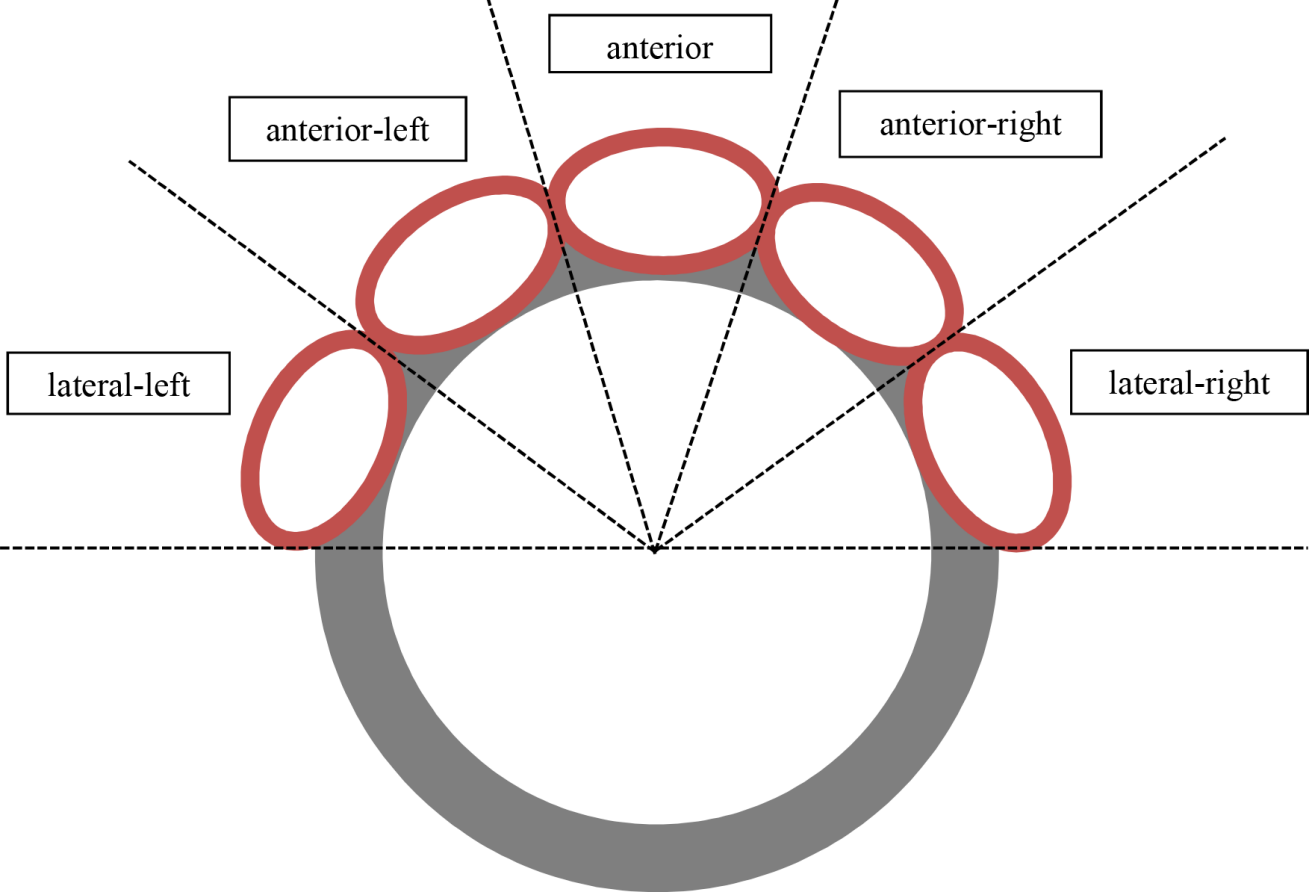
Ventricular relationships. Depicted is the morphologically left ventricle (grey circle) as seen from a superior position and the possible locations of the morphologically right ventricle (red ovals) along the anterior side of the morphologically left ventricle. The anterior side of the heart is divided in five parts (left/right and anterior/lateral) in intervals of 36°.

In this retrospective study, 39 DILV heart specimens from the National Heart & Lung Institute, Royal Brompton Hospital, Imperial College London, London, UK (n = 25) and the Leiden University Medical Center, Leiden University, Leiden, The Netherlands (n = 14) were examined. This study was conducted in accordance with both institutional guidelines for the use of human tissue. The specimen were consented to be studied under local ethics approval of aforementioned institutions. This study involved the use of donated hearts of children from the age of 0 years onwards. Due to the fact that the pathology to be studied is congenital in nature, including this vulnerable population was inevitable and essential for reliable data analysis. The authors had access to clinical information of individual donors during or after data collection. However, identification of individual donors was not possible because of the historic nature of the hearts and due to the fact that the hearts were donated anonymously, which means that the next of kind could not be asked to consent to inclusion in this study. Anatomically distorted hearts (i.e. hearts after Fontan completion or extensive post mortem dissection), hearts without two patent and separate atrioventricular valves, and fetal hearts were excluded. The hearts were analyzed in adherence with the methodology of the sequential segmental analysis of congenital heart disease [12]. Moreover, the spatial relationship of the ventricles to each other was examined. To determine the position of the heart in the ex vivo situation, the atrial septum was used as a reference point. The position of the morphologically right ventricle, as viewed in short axis cross-section, was categorized as being left or right as well as anterior or lateral in relation to the morphologically left ventricle. The distinction was made by dividing the anterior side of the heart in 5 segments and drawing lines at intervals of 36° on a template of the ventricular short axis (Fig 1).

Presence of relative outflow tract stenosis was estimated using the pulmonary trunk/aorta ratio. First, we measured the diameter of the narrowest point of the aortic and the pulmonary trunk orifices or outflow tract using Hegar dilators without expansion of the tissue. The cross-sectional areas a of the aorta and pulmonary trunk were calculated with a = πr2 (r: radius). The ratio of the diameter of the pulmonary trunk/aorta was considered abnormal if it was smaller than 1.05 or larger than 1.15 [13]. The ventricular septal defect (VSD) cross-sectional area was calculated using ImageJ® [14]. In order to avoid overestimation of the VSD diameter due to its noncircular shape, the effective diameter was calculated with the formula for the hydraulic diameter [15]. The VSD was considered to be restrictive in nature if its area was less than 70% of the diameter of the artery that depends on it. In hearts with discordant ventriculoarterial connection and in pulmonary atresia with the aorta originating from the morphologically right ventricle, the potential obstruction was towards the aorta. In hearts with concordant ventriculoarterial connection the potential obstruction was towards the pulmonary trunk. In hearts with a double outlet left ventricle, or aorta from the morphologically left ventricle and pulmonary atresia, or single outlet and common trunk originating from the morphologically left ventricle, the VSD does not have any effect in possibly restricting blood flow.

**Fig 2:**
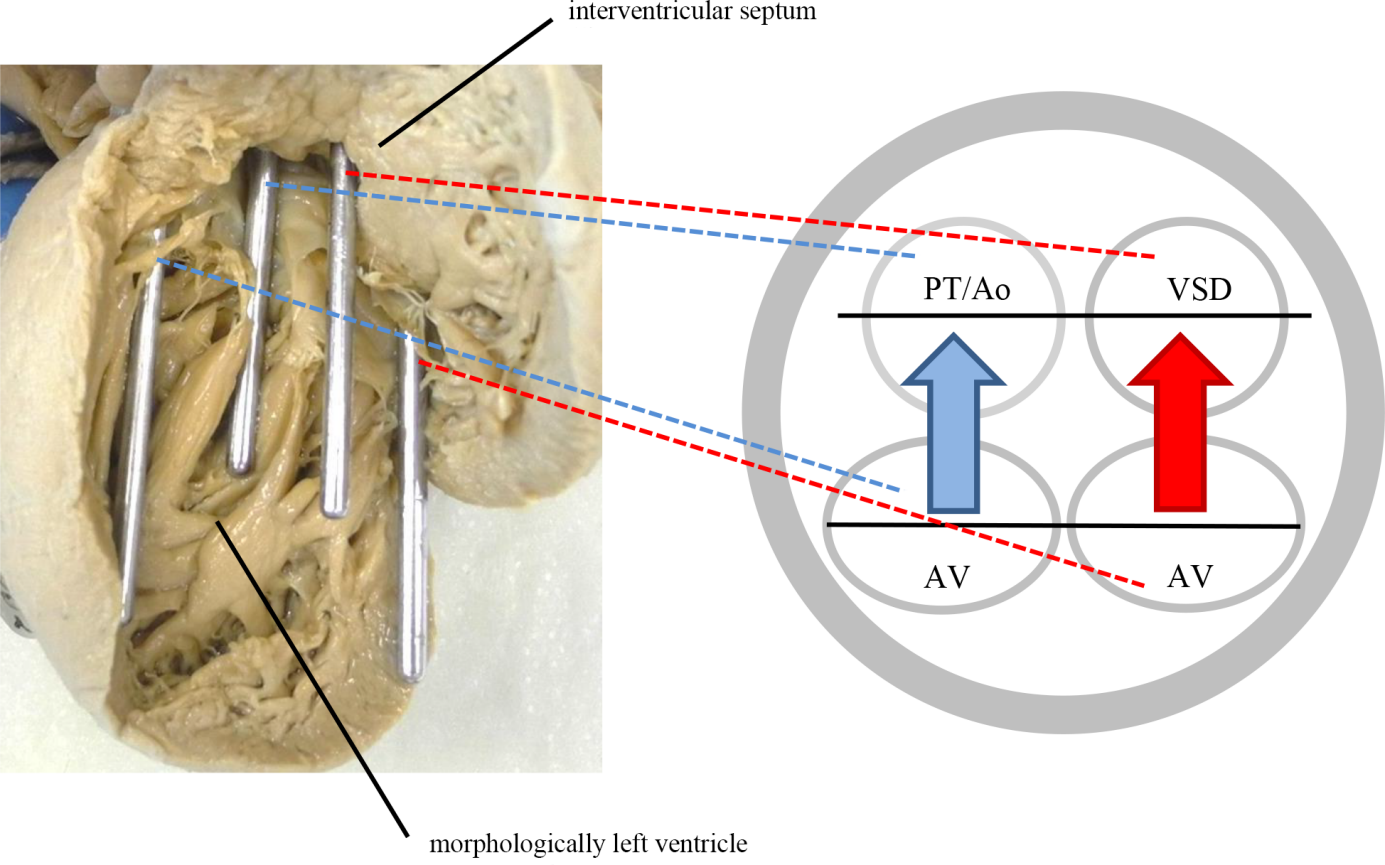
Parallel stand of the inflow and outflow tract planes (0°-10°). In this case, each of the outflow tracts is closer to one inflow tract, which leads to streaming and keeps the blood flows separated. The depicted heart has a discordant ventriculoarterial connection, meaning the pulmonary trunk is connected to the morphologically left ventricle and the aorta is connected to the morphologically right ventricle and communicates with the morphologically left ventricle via the ventricular septal defect. The angle in this specific heart was 5°.Ao: Aorta; AV: atrioventricular valve; PT: pulmonary trunk; VSD: ventricular septal defect; Ao = Aorta. Blue: deoxygenated blood, red: oxygenated blood.

**Fig 3:**
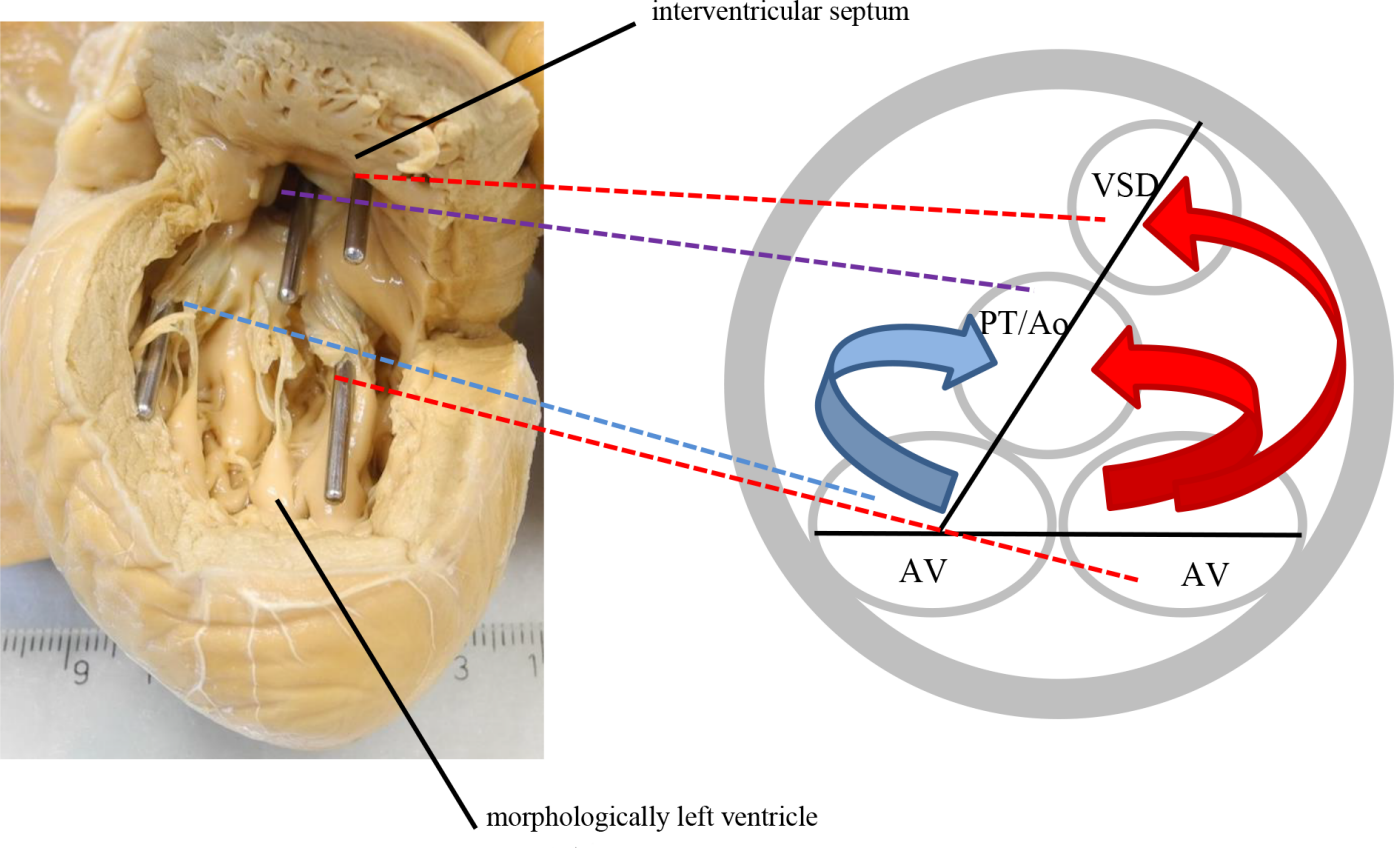
20°-70° angle of the inflow and outflow tract planes. One of the two outflow tracts is in closer proximity to both inflow tracts so that blood from both inflow tracts will enter this outflow tract, whereas the other outflow tract is only supplied by the one inflow tract it is closest to. The depicted heart has a discordant ventriculoarterial connection, meaning the pulmonary trunk is connected to the morphologically left ventricle and the aorta is connected to the morphologically right ventricle and communicates with the morphologically left ventricle via the ventricular septal defect. The angle in this specific heart was 45°.Ao: Aorta; AV: atrioventricular valve; PT: pulmonary trunk; VSD: ventricular septal defect; Ao = Aorta. Blue: deoxygenated blood, red: oxygenated blood, purple: mix of deoxygenated and oxygenated blood.

**Fig 4:**
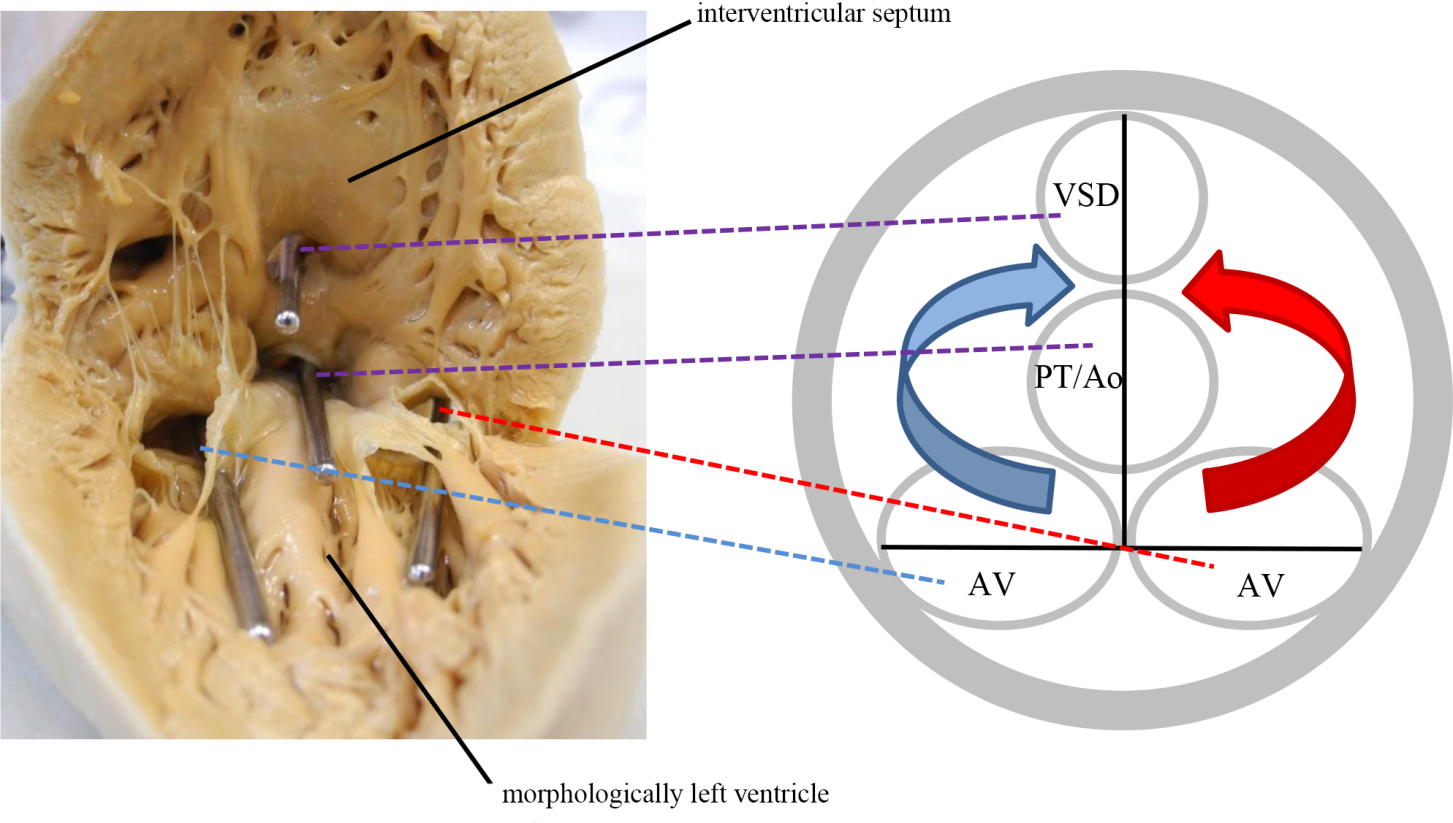
Perpendicular stand of the inflow and outflow tract planes (80°-90°). Blood from both inflow tracts enters both outflow tracts, leading to maximum mixture of deoxygenated and oxygenated blood. The depicted heart has a discordant ventriculoarterial connection, meaning the pulmonary trunk is connected to the morphologically left ventricle and the aorta is connected to the morphologically right ventricle and communicates with the morphologically left ventricle via the ventricular septal defect. The angle in this specific heart was 90°. Ao: Aorta; AV: atrioventricular valve; PT: pulmonary trunk; VSD: ventricular septal defect; Ao = Aorta. Blue: deoxygenated blood, red: oxygenated blood, purple: mix of deoxygenated and oxygenated blood.

The spatial relationship of the inflow and outflow tracts (i.e. the atrioventricular valves in relationship to the ventriculoarterial valves or to one ventriculoarterial valve and the VSD, if restrictive) was analyzed by inserting probes into the openings and drawing two lines connecting the two inflow tract openings and the two outflow tract openings. The angle at the intersection of the lines was measured and the hearts were then organized into three groups according to the angle. Firstly, the line connecting the atrioventricular valves ran almost parallel (0°-10°) to the line connecting the outflow tract orifices (Fig 2). Secondly, the in-between variant where the angles ranged from 20° to 70° **(**Fig 3**)**, and lastly, the arrangement of the lines was such that they formed a right angle (80°-90°) (Fig 4). A working model of possible blood flow patterns was created in correspondence with this spectrum. It was hypothesized to range from a relatively favorable flow pattern where blood flows might stay separated, to a less favorable flow pattern where both inlet flows would be directed to both outlets, potentially resulting in blood mixture or to a partially turbulent flow pattern with mild to moderate mixing (Figs 2–4).

## Results

### Patient characteristics

Data on sex and age of the patients was available in 28 out of 39 cases. Of those, 23 (82%) were male. Age at death ranged from 2 days to 39 years. The median age at death was 1 year 7 months (IQR 1.3 months– 15 years 7 months).

### Situs and atrioventricular connection

All DILV hearts had a situs solitus. In 37 hearts, both atrioventricular orifices were exclusively connected to the morphologically left ventricle. In 2 hearts, one of the atrioventricular orifices was overriding the ventricular septum and was thus connected to both ventricles. In 14 hearts, an interatrial communication was present at the oval fossa.

### Ventriculoarterial connection, spatial relationship of the ventricles and position of the great arteries

**Fig 5:**
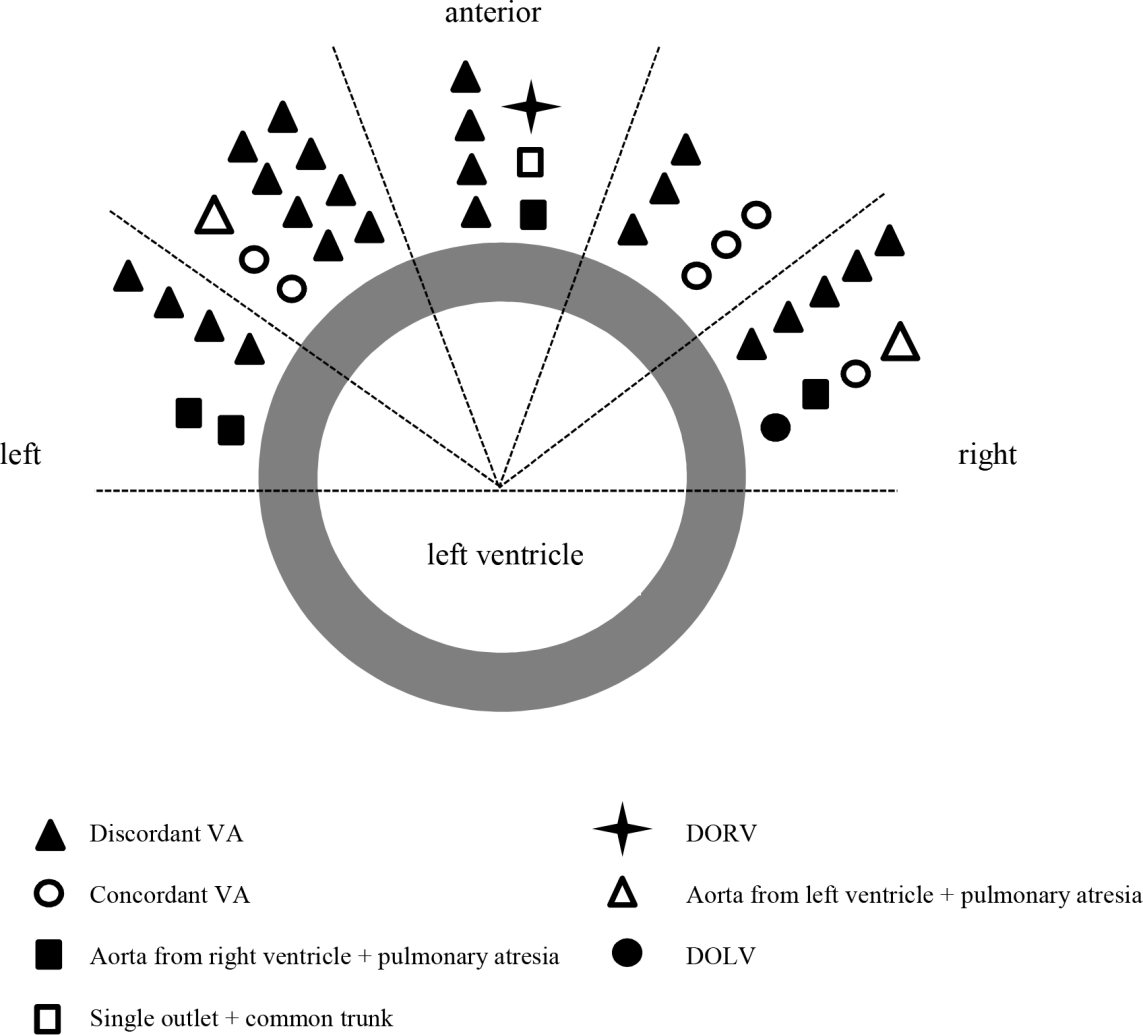
Distribution of the different types of DILV hearts. Hearts are arranged by their type of ventriculoarterial connection with the position of the morphologically right ventricle in relation to the morphologically left ventricle. DOLV: double outlet left ventricle, DORV: double outlet right ventricle, LV: morphologically left ventricle, RV: morphologically right ventricle, VA: ventriculoarterial connection.

The majority of specimens had a discordant ventriculoarterial connection with an anatomically left-sided morphologically right ventricle and a right anteriorly located aorta or a discordant ventriculoarterial connection, an anatomically right-sided morphologically right ventricle and a right anteriorly located aorta. Uncommon types were double outlet ventricles, pulmonary atresia, and common trunk (Table 1, Fig 5).

**Table 1:** Anatomical relationships of the right and left ventricle and the aorta andpulmonary trunk.

Ventriculoarterial connection (n)	Position of the RV	Position of the aorta
**Discordant (24)**	anterior (4) left (12) anterior-left (8) lateral-left (4) right (8) anterior-right (3) lateral-right (5)	anterior (1) anterior-left (1) anterior-right (2) anterior (2) anterior-left (2) anterior-right (4) anterior (1) anterior-left (2) anterior-right (1) anterior-left (2) anterior-right (1) anterior (1) anterior-right (4)
**Concordant (6)**	left (2) anterior-left (2) right (4) anterior-right (3) lateral-right (1)	posterior-right (2) anterior-left (1) posterior-left(2) posterior (1)
**Aorta from morphologically RV + valvar pulmonary atresia (4)**	anterior (1) left (2) lateral-left (2) right (1) lateral-right (1)	anterior (1) anterior (2) anterior (1)
**Double outlet right ventricle (1)**	anterior (1)	anterior-left (1)
**Aorta from morphologically LV + valvar pulmonary atresia (2)**	left (1) anterior-left (1) right (1) lateral-right (1)	anterior-left (1) anterior-left (1)
**Double outlet left ventricle (1)**	right (1) lateral-right (1)	anterior-right (1)
**Single outlet + common Trunk (1)**	anterior (1)	-

LV: Morphologically left ventricle; RV: Morphologically right ventricle.

### Presence of relative outflow tract stenosis

All but 2 hearts (93%) with 2 patent ventriculoarterial connections had a pulmonary trunk/aorta ratio outside the norm of 1.05–1.15 (Table 2).

**Table 2:** Pulmonary trunk-aorta ratios.

Ventriculoarterial connection (n)	PT/Ao ratio < 1.05	PT/Ao ratio 1.05–1.15	PT/Ao ratio > 1.15
**Discordant** (21)	5 (24%)	2 (9%)	14 (67%)
**Concordant** (5)	1 (20%)	-	4 (80%)
**DORV** (1)	1 (50%)	-	-
**DOLV** (1)	-	-	1 (100%)
**Single outlet** (1)	1 (100%)	-	-

Ao: Aorta; DOLV: double outlet left ventricle; DORV: double outlet right ventricle; PT: Pulmonary trunk.

Not included: hearts with valvar atresia (n = 6), hearts where measurement was technically not possible (n = 4).

### Ventricular septal defect

All 39 hearts had a VSD. The effective diameter of the VSD was less than 70% of the pulmonary orifice in 2 out of 5 (40%) hearts with a concordant connection (VSD diameter measurement technically not possible in 1 heart), in 10 out of 22 (45%) hearts with a discordant connection (VSD diameter measurement technically not possible in 2 hearts) and in 1 out of 4 (25%) hearts with pulmonary atresia and the aorta arising from the morphologically right ventricle. Furthermore, in the double outlet right ventricle, the effective diameter of the VSD was larger than 70% both for the aorta as well as the pulmonary orifice.

### Relationship of the inflow and outflow tracts

**Fig 6:**
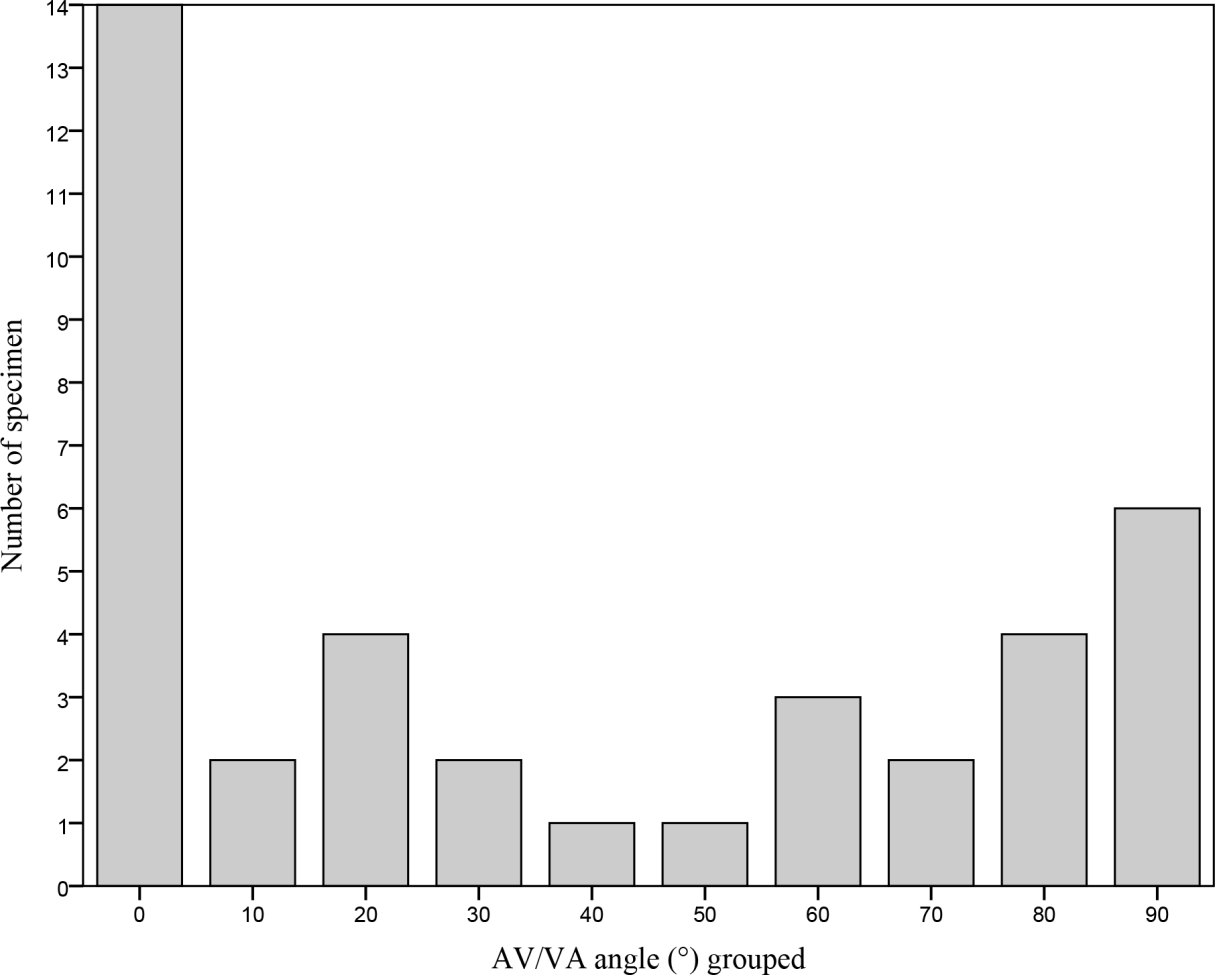
Distribution of the angles of the inflow and outflow tract planes. Depicted on the y axis are the number of specimen in each group, depicted on the x axis are the AV/VA angles, ranging from 0° to 90°, divided in 10 groups. AV: atrioventricular; VA: ventriculoarterial.

The largest group of hearts had an almost parallel position of the inflow and outflow tracts (Fig 2). The second largest group consisted of hearts with an almost perpendicular position of the inflow and outflow tracts (Fig 4). Furthermore, there was a fairly equal distribution with values between 20° and 70° (Fig 3). For quantification of the hearts see Fig 6.

## Discussion

The key finding of this study is that there is a spectrum of anatomic variants of DILV hearts with three potential flow patterns. Most hearts either had a parallel relationship of the inflow and outflow tract openings allowing for blood streams to remain separated, or the outflow tract was perpendicular with the inflow allowing for mixing. We are convinced that these findings are an important part of the anatomic basis of the streaming patterns of oxygenated and deoxygenated blood in patients surviving DILV without cavopulmonary connection. This study aimed to construct a hypothesis on how intracardiac anatomical characteristics may influence streaming and mixture of blood. This hypothesis may serve as a basis for future research using imaging modalities such as 4D MRI [16]. In line with the anatomical findings, we imply that a combination of parallel outflow tracts as well as moderate pulmonary stenosis would be the preferential for a DILV patient, that is purely hypothetically.

Fontan completion initially is associated with an increase in exercise capacity and decrease in cyanosis, allowing children with a functionally univentricular heart to live where they would have otherwise ultimately died. The long term results of the Fontan procedure, however, appear to be limited by the peculiarities of this iatrogenic and highly abnormal circulation; quality of life gradually decreases with an increasing age of the Fontan circulation. As a consequence, the experience and opinion of pediatric cardiologists in regards to the Fontan procedures diverges from that of adult congenital cardiologists [17].

With the long-term results of the Fontan circulation remaining compromised, serious considerations about improvements and alternatives might seem iconoclastic, but are definitely warranted. Since a small selected subgroup of DILV patients survives to old age without cavopulmonary connection, it is highly relevant to investigate what makes this group superior to Fontan patients regarding survival. Separation or mixing of oxygenated and deoxygenated intracardiac blood streams is for a large part determined by specific anatomical characteristics and relationships in functionally univentricular hearts [18]. This study describes these characteristics and relationships in post mortem DILV hearts without cavopulmonary connection, premising one of the basic principles of fluid dynamics. Streamlines, or laminar flows, such as they occur in the ventricle in the absence of obstruction, stay separated adjacent to each other [11]. In other words, they do not have a natural tendency to mix.

First attempts at hemodynamic characterization were already made by Rahimtoola in 1966, Macartney in 1976 and Mocellin in 1979 [18–20]. At that time the anatomical distinctions between the various forms of functionally univentricular hearts were not yet fully appreciated, so that results for DILV hearts cannot be explicitly extracted from these studies [21]. When considering epidemiological facts concerning functionally univentricular double inlet hearts, however, it becomes apparent that the double inlet ventricle of the left ventricular type is by far more common than that of the right, or indeterminate type [1]. This indicates that the majority of the hearts in the above named studies probably have been DILV hearts. It is therefore possible to deduce certain hemodynamic principles of functionally univentricular hearts from them. Macartney found that a lateral (both left and right) position of the “outlet chamber” (rudimentary right ventricle) was related to favorable streaming. The majority of our hearts in fact had a lateral position of the rudimentary morphologically right ventricle.

In addition to streaming, limitation of pulmonary blood flow constitutes the other part of this anatomic complex. Moderate pulmonary stenosis has a protective effect and is beneficial for balancing pulmonary and systemic flow and survival [22]. In almost our entire group of ex vivo heart specimens the ratio of the size of the great arteries was outside the norm, showing no evidence of pulmonary stenosis in most. The fact that pulmonary stenosis was uncommon might explain the non-viability of those hearts and could be due to the negative selection bias.

About half of the VSDs had a diameter that was less than 70% of the arteries’ diameter to which it gave access. In an in vivo setting, this could mean that the VSD was restrictive in nature, creating a sub-arterial stenosis. But this would be favorable in hearts with a concordant ventriculoarterial connection, since it would limit blood flow to the lungs. This was the case in only 2 out of 5 hearts. In hearts with a discordant ventriculoarterial connection, it could cause limitation of blood flow to the aorta. This was the case in about half of the ex vivo hearts, and is a well-recognized problem, that can be solved clinically by enlarging the VSD surgically [23].

If in DILV hearts the atria are separated, the pulmonary and systemic blood streams enter the morphologically left ventricle in parallel during diastole. Analysis of the relationship of the inflow and outflow tracts showed that in a majority of the hearts, the angle of the line connecting the two atrioventricular valves and the two outflow tracts (two ventriculoarterial valves or one ventriculoarterial valve and the VSD) was rather small. This means that the inflow tracts were more or less directly opposite the outflow tracts. This might have resulted in a favorable, linear direction of blood streams in vivo and provided the highest chance of keeping the blood streams separated. At the other end of the spectrum were hearts with an almost right angular relationship of the inflow and outflow tracts. In this anatomical variant, one of the outflow tract openings was considerably closer to both inflow tract openings. In vivo, this difference might have facilitated intraventricular mixing of oxygenated and deoxygenated blood due to deviating, non-linear blood streams. This might explain why in this study, a relatively small proportion of these patients survive into adulthood without surgery except pulmonary artery banding.

It is interesting to consider a species that naturally possesses a single ventricle: deoxygenated and oxygenated blood stay separated in the univentricular frog heart [24]. As in DILV hearts, frog hearts have two atrial chambers, which are connected to the ventricle by two atrioventricular orifices [25]. The ventricle discharges into a single contractile conus arteriosus before being distributed into the pulmonary and systemic arteries by a spiral fold [26]. Moreover, pulmonary and arterial pressures in the frog are approximately equal, whereas pulmonary and systemic resistance can be adjusted, all in all obviating the need for limiting pulmonary blood flow by stenosis [27]. In human DILV hearts a well-balanced size of the two separate outflow tracts is crucial; a sufficiently large aorta and a moderately stenotic pulmonary trunk would facilitate more or less equal pulmonary and systemic flows, resulting in an arterial oxygen saturation that is compatible with life, without risk of pulmonary hypertension [28]. In the frog heart, blood from the right atrium stays on the right side of the ventricle and is directed towards the conus arteriosus by direct alignment and close proximity with the base of the conus arteriosus. This alignment, when present, might also contribute to limiting the mixture in the human heart. A parallel position of the inflow and outflow tracts as described above might best represent this scenario (Fig 2).

Uemura et al investigated the morphologic spectrum of a series of post-mortem DILV specimen as well as of clinical patients with DILV [29]. Hearts with the hypoplastic morphologically right ventricle on the left side were the largest group in the current study as well as in the study by Uemura, although the overall percentage of these was considerably lower in our study. In our study, however, a relatively large number of DILV hearts showed a wide range of anatomical heterogeneity. This difference might be explained by the fact that in Uemura’s study, as opposed to ours, half of the hearts included were living DILV patients with 80% having a discordant ventriculoarterial connection, which might have had more favorable blood streams, allowing them to survive.

The setting of an ex vivo study brings limitations: The hemodynamic implications of this study are based on theoretical assumptions. However, despite the lack of direct clinical relationships, this study is of utmost importance since it gives an overview of the anatomic variability of DILV hearts and the possible relationship between the anatomy and physiology. The hearts were independent of any anatomical extracardiac structures that would normally determine their position in vivo. In this study, the atrial septum was therefore regarded as the reference point. Due to a lack of patient data, it was not possible to make any correlations with clinical parameters, such as body surface area, in order to determine valve size abnormalities, or to take physiological characteristics, such as pulmonary vascular resistance, which plays a major role in the distribution of blood flows, into account. Stenosis and restriction are terms used in relation to a circulating blood flow in vivo and their presence is determined in a beating heart. In this study, ratios were used to quantify abnormalities in size. This study has an inherent selection bias: Most of the ex vivo heart specimens were small hearts suggesting that the patients died rather young. The individual reasons why the patients did not undergo surgery are unknown, but most of the ex vivo hearts were from an era where no adequate surgical procedure had yet been established for the palliation of those patients. The hearts being unable to sustain sufficient circulation due to unfavorable anatomy could explain their early death. In half of the hearts in this study, a defect in the oval fossa might have facilitated some, albeit limited, mixing of atrial blood. Since our study group only had hearts with two separate atrioventricular orifices, which in vivo would have guaranteed two independent, separated streams into the ventricle, the effect of possible atrial blood mixture would be negligible.

## Conclusions

The data on patients with unoperated DILV hearts living up to adulthood without major symptoms are proof that there are certain anatomical features within the heart that are compatible with longer term survival. It was possible to quantify the hearts according to the type of the ventriculoarterial connection, the location of the rudimentary morphologically right ventricle in relation to the morphologically left ventricle, the ventricular septal defect and the position of the aorta in relation to the pulmonary trunk. In this study, the most common variants were DILV hearts with a discordant ventriculoarterial connection, the morphologically right ventricle to the left and the aorta right anterior. The relationship of the inflow and outflow tracts probably plays a crucial role in keeping the blood streams separated in vivo. Many factors regarding the mechanism of blood flow separation in DILV hearts are still unknown. Nevertheless, the poor long-term results of the Fontan circulation and the data on natural survivors do raise important considerations regarding treatment approach to certain patients with a single ventricle diagnosis. For a subgroup of patients, therapeutic nihilism or an alternative surgical procedure, such as pulmonary banding to protect the pulmonary vasculature from overcirculation, might increase life expectancy and quality of life. This study lays the ground for establishing a theoretical relationship between anatomy and physiology in this difficult and heterogeneous patient group. Further research into the mechanisms of blood flow in an in vivo setting is imperative.

## Supporting information

S1 File Supporting data. This is the minimal data set, including all data used for the analysis of this paper.
(XLSX)
